# 죽음인식, 임종간호 스트레스, 삶의 만족도가 상급종합병원 간호사의 임종간호태도에 미치는 영향

**DOI:** 10.7586/jkbns.24.019

**Published:** 2024-08-23

**Authors:** Yoon Young Shin, Haejung Lee

**Affiliations:** 1 Pusan National University Hospital, Busan, Korea; 1 부산대학교병원; 2 College of Nursing, Pusan National University, Yangsan, Korea; 2 부산대학교 간호대학; 3 Research Institute of Nursing Science, Pusan National University, Yangsan, Korea; 3 부산대학교 간호과학연구소

**Keywords:** Terminal care, Attitude to death, Personal satisfaction, 임종간호, 임종태도, 삶의 만족도

## Abstract

**Purpose:**

This study investigated the factors influencing attitudes toward end-of-life care among nurses at a tertiary hospital in Korea. Specifically, it examined the roles of nurses’ characteristics, death perceptions, terminal care stress, and life satisfaction.

**Methods:**

The participants included 150 nurses working at a tertiary hospital, who were recruited between August 15 and September 19, 2023. Data were collected through an online survey and analyzed using descriptive statistics, the t-test, analysis of variance, Pearson correlation coefficients, and multiple regression analysis with SPSS/WIN 28.0.

**Results:**

Nurses’ attitudes toward end-of-life care were positively correlated with death perceptions (β = .28, *p* < .001), 3 years or more of clinical experience (β = .25, *p* = .001), the experience of an acquaintance death (β = .22, *p* = .002) and life satisfaction (β = .20, *p* = .004). These variables explained 34.0% of the total variance in attitudes toward end-of-life care.

**Conclusion:**

It is essential to develop and implement individualized end-of-life nursing education programs, particularly utilizing simulations, for nurses with limited clinical experience and low levels of death perceptions. Further research should explore attitudes toward end-of-life care among various healthcare providers with a broader regional scope to improve the overall quality of end-of-life care.

## 서론

### 1. 연구의 필요성

죽음은 성장의 마지막 단계이고 누구도 피할 수 없는 과정이며, 인간 존재에 의미를 부여하는 우리 삶의 필수적인 부분이다[1]. 의학기술 발달로 인한 수명 연장과 급속한 인구 고령화 현상, 만성 질환자의 증가, 가족구조의 변화 등으로 의료기관에서 임종을 맞이하는 환자 수가 증가하고 있다[2-4]. 이에 따라 임종환자를 가장 가까이에서 돌보며 죽음을 잘 받아들일 수 있도록 돕는 간호사의 임종간호에 대한 중요성이 강조되고 있다[2]. 한편 상급종합병원은 급성기 중증질환에 대한 의료서비스를 제공하고, 치사율이 높거나 진단 난이도가 높은 질병을 다루며[5], 다양한 치료단계가 혼합되어 있고 업무 속도가 빠른 환경적 특성으로 임종을 앞둔 말기 환자 치료에 대한 우선순위가 비교적 낮게 다루어지는 경향이 있다[6,7]. 상급종합병원에서의 말기 환자 치료 시 주된 목표는 호스피스 병동이나 요양원으로 의뢰하는 것이지만[8], 2024년 7월 기준 상급종합병원 47개 중 입원형 호스피스 서비스를 제공하는 병원은 18개뿐으로 그 수가 매우 부족하여[9], 대부분의 임종은 일반병동이나 중환자실에서 이루어진다[10]. 이에 따라 상급종합병원 간호사들은 과중한 업무량으로 임종간호에 충분한 지원과 시간을 투자하지 못하고, 말기환자와 가족들에게 성실하고 인간적인 간호를 제공하고자 하는 간호사의 노력이 방해받기도 하며, 높은 스트레스와 열정 부족, 죄책감, 우울증, 회의감 등의 고통을 느끼는 것으로 보고된다[8,10,11]. 이러한 어려운 상황 속에서도 간호사들은 임박한 죽음과 관련된 증상을 관리하기 위해 계획을 세우고, 임종환자와 가장 많은 시간을 보내며 신체적, 정신적 지지를 제공[11]하는 중요한 역할을 한다.

임종간호는 삶의 종말에 다다른 환자와 가족을 위해 간호사가 보살핌을 제공하는 것으로 정의된다[12]. 임종환자 간호에 대해 간호사는 긍정적 또는 부정적 감정과 인식을 가지며, 감정과 인식은 태도로 나타난다[2,10]. 임종간호에 대한 인식이 긍정적일수록 연명의료 관련 법적 제도에 대한 지식이 높고, 통증을 포함한 임종 전 증상에 대해 신체적 돌봄과 약물적 중재를 적극적으로 제공한다[11,13]. 또한, 임종 환자가 존엄성을 유지하면서 편안한 죽음을 맞이할 수 있도록 돕고, 가족들이 죽음을 수용하고 더 성숙한 인간으로 발전할 수 있도록 돕는다[14]. 긍정적 임종간호태도를 가진 간호사는 임종상황에서 삶의 의미와 가치를 깨닫고[15], 높은 공감능력으로 타인의 고통을 공감하며 적극적으로 임종간호를 수행한다[14].

임종간호태도는 죽음인식[16-18], 임종간호 스트레스[16,17,19], 삶의 만족도[16,20,21], 연령[16,17,22], 결혼상태[16], 종교[20], 최종학력[17,20], 총 임상경력[15,23], 최근 1년 이내 가족이나 지인의 죽음경험[22,24], 임종간호 횟수[16,22,24], 임종관련 교육경험[14,18,24] 등 다양한 간호사 특성과 관련이 있는 것으로 보고되었다. 죽음인식은 개인이 죽음에 대해서 느끼는 감정과 죽음에 대한 인지, 개인적인 신념을 포함하는 복합적인 개념으로, 개인의 가치관과 경험과 관련된다[18]. 간호사가 죽음을 자연스러운 과정으로 인식하면 임종환자의 죽음에 대한 불안도를 낮추고 죽음을 수용하게 도울 수 있다[16]. 또한 간호사가 죽음에 대한 관심과 호기심이 높고[18], 긍정적 죽음 인식을 가질 때, 긍정적 임종간호태도를 나타낼 수 있다[25].

임종환자를 돌보는 것은 간호사에게 감정적으로나 육체적으로 힘든 일이고 높은 스트레스 요인일 수 있다[17]. 임종간호 스트레스는 임종환자를 돌보는 과정에서 죽음에 대한 무력감, 피로감, 보상이 없는 헌신에 따른 절망감, 고립감과 함께 느끼는 심리적 고통 등을 포함한다[16,26]. 신체적, 심리적, 영적인 측면에서 임종환자의 많은 요구들이 충족되지 못하고, 임종간호의 효과가 부족하다고 생각될 때 간호사는 높은 임종간호 스트레스를 경험한다[27]. 높은 임종간호 스트레스는 간호사들이 임종환자를 돌보는데 소극적인 태도를 취하게 하고[28], 임종환자가 존엄성을 유지하고 편안한 죽음을 맞이할 수 있도록 돕는 임종간호를 방해한다[16,29].

간호사 개인의 삶의 만족도도 임종간호태도에 영향을 미칠 수 있다. 간호사 자신이 행복하고 인간다운 삶을 영위하고 개인의 삶을 만족하면 간호대상자의 삶의 질을 높이는 방향으로 간호를 제공하는 경향이 높을 수 있다. 삶의 만족도가 높은 간호사는 생명존중에 대한 의지가 높고 죽음에 대한 부정적 인식이 낮으며[30], 긍정적인 임종간호태도를 나타낼 수 있다. 특히 밀레니얼 세대와 Z세대를 통합한 MZ세대는 직무만족도가 낮고 이직의도가 높으며[31], 삶과 일의 균형을 중요시한다. 상급종합병원 인력의 80%는 만 39세 이하 간호사로 MZ세대에 해당하며[32], MZ세대의 특성을 고려해 볼 때 개인의 삶의 만족도는 임종간호태도에 영향을 미칠 수 있다. 그러나 임종간호태도 관련 대부분 연구는 2010년대에 수행되어, 개인의 삶의 만족도와 임종간호태도와의 관련성을 조사한 연구는 찾아보기 어렵다. 따라서 MZ세대가 간호사 그룹의 주력세대로 역할하고 있는 현시점에서 간호사 개인의 삶의 만족도와 임종간호태도와의 관련성을 확인하여 이들의 긍정적인 임종간호태도 함양을 위한 연구의 토대를 마련할 필요가 있다.

상급종합병원의 간호사를 대상으로 한 기존 연구들은 죽음불안, 임종간호 스트레스, 임종간호수행 간의 관련성[2], 의사와 간호사가 경험하는 임종간호관련 도덕적 딜레마[8], 스트레스와 직무수행 간의 관계에서 감정이입과 탄력성의 매개효과[10], 임종간호인식과 수행[11]에 대해 조사하여, 임종간호에서 중요한 변수인 임종간호태도에 대한 연구는 부족하다. 또한 간호사 특성, 죽음인식, 임종스트레스와 함께 간호사 개인의 삶의 만족도가 임종간호태도에 미치는 영향을 분석한 연구는 거의 없다. 이에 본 연구는 상급종합병원 간호사의 일반적 특성과 죽음인식, 임종간호 스트레스, 삶의 만족도 중 임종간호태도와 가장 관련성이 높은 특성을 파악하여 추후 간호사의 임종간호태도 향상을 위한 전략 개발의 기초자료를 제공하고자 한다. 이는 임종환자와 가족에게 더 나은 의료 서비스를 제공할 수 있는 기초자료 마련과 간호사의 임종간호 수행을 향상하기 위한 시스템 개발에 중요한 근거를 제공할 것으로 기대된다.

### 2. 연구 목적

본 연구의 목적은 상급종합병원 간호사의 죽음인식, 임종간호 스트레스, 삶의 만족도 및 임종간호태도를 파악하고, 임종간호태도의 영향요인을 확인하는 것이다. 구체적인 목적은 다음과 같다.

1) 상급종합병원 간호사의 죽음인식, 임종간호 스트레스, 삶의 만족도 및 임종간호태도 정도를 파악한다.

2) 상급종합병원 간호사의 일반적 특성에 따른 임종간호태도를 파악한다.

3) 상급종합병원 간호사의 죽음인식, 임종간호 스트레스, 삶의 만족도 및 임종간호태도 간의 상관관계를 분석한다.

4) 상급종합병원 간호사의 임종간호태도 영향요인을 규명한다.

## 연구 방법

### 1. 연구 설계

본 연구는 상급종합병원 간호사의 죽음인식, 임종간호 스트레스, 삶의 만족도 및 임종간호태도를 파악하고 임종간호태도의 영향요인을 확인하기 위해 수행한 서술적 조사 연구이다.

### 2. 연구 대상

본 연구의 대상자는 부산시에 소재하는 상급종합병원인 부산대학교병원에서 호스피스 병동을 제외한 일반병동이나 중환자실에서 근무하고 있고, 임종환자를 1회 이상 간호한 경험이 있는 간호사이다. 선행연구를 참고하여[17] 독자적으로 임종간호를 제공하는 것에 어려움이 있을 수 있는 1년 미만의 신규 간호사는 제외하였다. 직접 환자간호를 제공하지 않는 수간호사 이상의 관리자급 간호사와 임종간호에 직접 참여하지 않는 외래, 수술실, 응급실 등 특수파트에서 근무하는 간호사는 제외하였다.

본 연구에 필요한 표본크기는 G*Power 3.1.9.7 프로그램(University of Dusseldorf, Dusseldorf, Germany)을 이용하여 산출하였다. 선행연구[17]에 근거한 효과크기(d) .15, 유의수준(α) .05, 검정력(1-β) 0.8, 일반적 특성 및 연구변수 14개를 기준으로 다중회귀분석에 필요한 표본 수는 최소 135명으로 산출되었다. 본 연구에서는 불성실한 응답률 15%를 고려하여 159명에게 설문을 수행하였으며 호스피스병동에 근무한다고 응답한 9명의 응답자를 제외한 총 150명의 설문 응답을 최종 분석에 사용하였다.

### 3. 연구 도구

#### 1) 일반적 특성

본 연구에서 연구대상자의 일반적 특성은 간호사의 임종간호태도 영향요인을 조사한 선행연구를 참고하여[17] 성별, 연령, 결혼상태, 종교, 최종학력, 총 임상경력, 현 근무부서, 최근 1년 이내 가족이나 지인의 죽음경험, 임종간호 횟수, 임종관련 교육경험으로 총 10가지 항목으로 조사하였다.

#### 2) 죽음인식

죽음인식은 ‘죽음과의 관계에서 바라본 삶에 대한 태도’로 본 연구에서는 Cha [30]가 간호사를 대상으로 개발한 도구를 사용하여 측정하였다. 본 도구는 36 문항, 5개 하위영역으로 구성되며, 하위영역은 죽음에 대한 긍정도 10 문항, 죽음에 대한 부정도 10 문항, 죽음에 대한 불안도 4 문항, 죽음에 대한 관심도 6 문항, 생명존중의지 6 문항이다. 모든 문항은 ‘매우 그렇지 않다’ 1점에서 ‘매우 그렇다’ 5점까지의 5점 Likert 척도로 측정하였으며, 점수가 높을수록 죽음에 대한 인식 정도가 높은 것을 의미한다. 본 도구의 신뢰도 Cronbach's α는 Cha [30]의 연구에서 .79이었고, 본 연구에서는 .81이었다.

#### 3) 임종간호 스트레스

임종간호 스트레스는 Lee [27]가 개발한 임종간호 스트레스 도구를 사용하여 측정하였다. 도구는 총 40문항, 7개의 하위영역으로 구성되며, 하위영역에는 ‘간호사의 전문지식과 기술부족’, ‘의료한계에 대한 갈등’, ‘간호사의 업무량 과중’, ‘임종환자에 대한 간호시간 할애의 어려움’, ‘임종환자 간호에 대한 어려움’, ‘임종환자와의 인간적 갈등’, ‘환자 및 보호자가 임종에 대해 가지는 부정적인 태도’가 포함된다. 모든 문항은 ‘매우 아니다’ 1점부터 ‘매우 그렇다’ 5점까지의 5점 Likert 척도로 측정하였으며, 점수가 높을수록 임종간호에 대한 스트레스 정도가 높은 것을 의미한다. 본 도구의 신뢰도 Cronbach’s α는 Lee [27]의 연구에서 .93이었고, 본 연구에서는 .98이었다.

#### 4) 삶의 만족도

삶의 만족도는 사람들이 자신의 삶 전체를 어떻게 평가하는지에 대한 측정으로, 삶에 대한 주관적인 경험과 감정을 의미한다[33]. 직무 만족도 측정 시 단일 질문 도구를 사용했을 때 복잡한 개념을 간결하고 명확하게 포착하여 평가 시간이 단축되고 복잡한 다중 질문 방식과 유의미한 상관관계를 보였다는 선행연구[34]를 참고하여 본 연구에서는 삶의 만족도 측정을 위해 ‘귀하의 삶의 만족도는 어떻게 됩니까?’ 라는 단일 질문 도구를 이용하여 평가하였다. 반응범위는 ‘매우 불만족’ 1점부터 ‘매우 만족’ 5점까지이었고 점수가 높을수록 삶의 만족도가 높은 것을 의미한다.

#### 5) 임종간호태도

임종간호태도란 임종을 앞둔 환자와 가족에게 신체, 정서, 사회, 영적 간호를 제공하는 임종간호에 대해 간호사가 가진 호의적 또는 비호의적인 반응을 일관성 있게 나타내는 복합적 태도이다. 임종간호태도는 Frommelt [35]의 Frommelt Attitudes toward Nursing Care of the Dying Scale을 Cho와 Kim [36]이 번역․수정․보완한 도구로 측정하였다. 긍정적 서술 15 문항과 부정적 서술 15 문항으로 총 30문항으로 구성되며 점수가 높을수록 임종간호태도가 긍정적인 것을 의미한다. 모든 문항은 ‘매우 그렇지 않다’ 1점부터 ‘매우 그렇다’ 4점까지 4점 Likert 척도로 측정하였으며, 부정문항은 역으로 점수를 환산하였다. 신뢰도 Cronbach’s α는 개발 당시 Frommelt [35]의 연구에서는 .94이었고, 한국어판 도구를 사용한 Cho와 Kim [36]의 연구에서는 .86, 본 연구에서는 .80이었다.

### 4. 자료 수집

자료수집은 2023년 8월 15일부터 2023년 9월 19일까지 이루어졌으며, 부산대학교병원 간호부에 연구의 취지를 설명하고 자료수집에 대한 승인을 받은 후 진행하였다. 외래와 수술실, 응급실 등 특수파트를 제외한 병동 간호사실 게시판에 연구의 목적, 연구내용, 절차, 자료의 활용에 대한 설명과 온라인 설문 접속을 위한 URL 및 QR코드가 첨부된 안내문을 부착하여 연구대상자를 모집하였다. 연구에 자발적으로 참여한 간호사는 온라인상에서 연구참여 및 자료 활용에 대한 동의서를 작성한 후 설문을 진행하도록 하였으며, 연구참여에 동의하지 않으면 설문이 진행되지 않도록 설정하였다. 설문 응답은 언제든지 중단이 가능하며, 모든 설문을 작성한 후 제출 버튼을 누르지 않으면, 중단한 설문자료는 저장되지 않도록 하였다.

### 5. 자료 분석

본 연구에서 수집된 자료는 SPSS/WIN 28.0 통계 프로그램(IBM Corp., Armonk, NY, USA)을 이용하여 분석하였으며 구체적인 자료분석 방법은 다음과 같다.

1) 대상자의 일반적 특성은 실수와 백분율로, 죽음인식, 임종간호 스트레스, 삶의 만족도 및 임종간호태도의 정도는 평균과 표준편차로 분석하였다.

2) 대상자 특성별 임종간호태도 평균차이 검증은 t-test와 one-way ANOVA로 하였고, 사후 분석은 Scheffé test를 이용하여 검증하였다.

3) 죽음인식, 임종간호 스트레스, 삶의 만족도 및 임종간호태도 간의 상관관계는 Pearson correlation coefficients를 이용하여 분석하였다.

4) 임종간호태도 영향요인은 동시적 다중회귀분석(simultaneous multiple regression)을 이용하여 분석하였다. 다중회귀분석식에 포함된 예측인자는 단변량 분석에서 임종간호태도와 유의한 관련성이 있는 것으로 나타난 일반적 특성(총 임상경력, 최근 1년 이내 가족이나 지인의 죽음경험, 임종관련 교육경험)과 삶의 만족도, 죽음인식이다. 일반적 특성인 총 임상경력, 최근 1년 이내 가족이나 지인의 죽음경험, 임종관련 교육경험은 가변수(dummy variable) 처리하여 회귀식에 포함하였고, 임상경력은 사후검증결과에 근거하여 ‘3년 이상’을 ‘1’, 나머지를 ‘0’으로 더미 코딩하여 회귀식에 포함하였다.

### 6. 윤리적 고려

본 연구는 연구대상자들의 권리와 윤리적 보호를 위하여 자료수집 전 부산대학교의 연구윤리심의위원회(IRB)의 승인을 받은 후 진행하였다(IRB No.2023_93_HR). 연구참여자 모집을 위해 연구목적, 연구절차 및 방법, 익명성 보장, 온라인 설문지 접속 방법(URL, QR코드)이 기재된 안내문을 게시하여 자발적으로 설문조사에 참여하도록 하였다.

온라인 설문조사서에는 연구의 목적, 절차 및 익명성 보장, 중단 시 불이익이 없음, 자료 활용 등에 대한 설명문을 게시하였고, 설문조사 참여에 동의를 표한 경우에 설문이 진행되고, 동의하지 않음을 표시하면 즉각적으로 조사가 중단되도록 설계하였다. 설문작성에 소요된 시간은 약 20분 정도이었고, 설문참여자에게는 설문 종료 후 소정의 상품권을 모바일로 전송하였다.

수집된 자료는 학술적 통계목적으로만 사용하였고, 연구자만 접속 가능한 비밀번호를 설정한 컴퓨터에 보관하였다. 외부의 접근을 제한하도록 방화벽을 설정하고 주기적으로 서버의 안전성을 점검하였다. 자료는 연구 종료 후 3년간 보관 후 복원 불가능한 방법으로 전자 파일을 삭제할 것이다.

## 연구 결과

### 1. 연구대상자의 일반적 특성, 죽음인식, 임종간호 스트레스, 삶의 만족도 및 임종간호태도

본 연구에 참여한 간호사(N = 150) 중 129명(86.0%)이 여성이었고, 평균연령은 29.91 ± 3.71세이었고, 20∼29세가 78명(52.0%), 30∼39세가 69명(46.0%)이었다. 미혼인 대상자가 100명(66.7%)이었고, 108명(72.0%)이 종교가 없다고 응답하였다. 최종학력은 학사졸업이 122명(81.3%)으로 대부분을 차지하였고, 총 임상경력은 7∼10년 미만이 41명(27.3%), 5∼7년 미만이 34명(22.8%), 3∼5년 미만이 32명(21.3%), 1∼3년 미만이 23명(15.3%), 10년 이상이 20명(13.3%)이었다. 현재 근무부서는 병동이 106명(70.7%), 중환자실이 44명(29.3%)이었고, 최근 1년 이내 가족이나 지인의 죽음을 경험한 대상자는 41명(27.3%)이었고, 임종관련 교육경험이 있는 대상자는 53명(35.3%)이었다. 1년 이내 임종간호 횟수는 5회 미만이 83명(55.3%) 이었다(Table 1). 대상자의 죽음인식 정도는 평균 108.69 ± 13.14이었고, 임종간호 스트레스는 평균 153.14 ± 30.36점이었다. 삶의 만족도는 평균 3.25 ± 0.72점이었고, 임종간호태도는 총점 85.80 ± 8.40점이었다(Table 2).

### 2. 연구대상자의 일반적 특성에 따른 임종간호태도

본 연구에 참여한 간호사의 일반적 특성에 따른 임종간호태도는 총 임상경력(F = 4.89, *p* = .001), 최근 1년 이내 가족이나 지인의 죽음경험(t = 3.61, *p* < .001), 임종관련 교육경험(t = 3.81, *p* < .001)에 따라 유의한 차이가 있었다. 총 임상경력에 대한 사후 검증 결과, 임상 근무경력이 1∼3년 미만인 경우보다 3년 이상인 경우에서 임종간호태도가 유의미하게 높은 것으로 나타나, 총 임상경력이 3년 이상이고, 최근 1년 이내 가족이나 지인의 죽음을 경험하였고, 임종관련 교육경험이 있는 경우, 임종간호태도 점수가 높았다(Table 1).

### 3. 연구대상자의 죽음인식, 임종간호 스트레스, 삶의 만족도 및 임종간호태도 간의 상관관계

죽음인식과 임종간호 스트레스, 삶의 만족도, 임종간호태도 간의 상관성을 파악하기 위해 Pearson 상관분석을 실시한 결과, 임종간호태도는 죽음인식(r = .41, *p* < .001)과 삶의 만족도(r = .24, *p* = .003)와 유의한 정적 상관성이 있었고, 임종간호 스트레스와는 유의한 상관성이 없는 것으로 나타났다(Table 3).

### 4. 임종간호태도에 영향을 미치는 요인

다중회귀분석 전 다중회귀분석을 위한 가정검증을 실시한 결과, 회귀모형의 적합성은 충족되었고(F = 15.95, *p* < .001), 공차(tolerance)는 .89∼.98으로 0.1 이상이었고, 분산팽창인자(Variance Inflation Factor)는 1.03∼1.12로 기준치인 10을 초과하지 않아 다중공선성의 문제가 없는 것으로 확인되었다. Durbin-Watson 통계량은 1.45로 자기상관위험은 없는 것으로 나타났다. 잔차 통계분석에서 표준화 잔차의 범위가 2.19∼4.02로 2에 근접하여 등분산성을 만족하였다.

다중회귀분석 결과, 죽음인식, 3년 이상의 임상경력, 최근 1년 이내 가족이나 지인의 죽음경험, 삶의 만족도, 임종관련 교육경험이 임종간호태도에 영향을 미치는 주요 요인으로 나타났으며, 이들 영향요인들은 임종간호태도 변량을 34.0% 설명하였다. 즉 죽음인식 점수가 높고(β= .28, *p* < .001), 임상경력이 3년 이상이고(β = .25, *p* < .001), 최근 1년 이내 가족이나 지인의 죽음을 경험하였고(β = .22, *p* = .002), 삶의 만족도가 높고(β = .20, *p* = .004), 임종관련 교육경험이 있을 때(β = .16, *p* = .030), 임종간호태도 점수가 높았다(Table 4).

## 논의

본 연구는 상급종합병원 간호사의 죽음인식, 임종간호 스트레스, 삶의 만족도 및 임종간호태도를 확인하고, 상급종합병원 간호사의 임종간호태도에 영향을 미치는 요인을 분석하여, 임종간호태도 향상을 위한 프로그램 개발의 근거 자료로 활용하고자 시행하였다. 본 연구에 참여한 간호사는 여성이 129명(86.0%)으로 대부분이었으며, 연령은 20∼29세가 78명(52.0%), 30∼39세가 69명(46.0%)이었다. 이러한 일반적 특성은 상급종합병원 간호사의 대부분이 여성(94.9%)이며 29세 이하가 46.3%, 30세에서 39세 이하가 33.2%라는 보건의료인력실태조사 결과[32]와 비교하였을 때, 본 연구에 포함된 간호사의 여성의 비율이 낮고(94.9% vs. 86.0%), 전체 참여 간호사 대비 20∼29세(46.3% vs. 52.0%)와 30∼39세(33.2% vs. 46.0%)의 간호사 분포가 높은 경향이 있어 본 연구결과의 확대해석에 주의가 필요하다.

본 연구에 참여한 간호사의 임종간호태도는 총점 85.80점으로 동일한 도구를 사용한 선행연구에서 중환자실 간호사 87.46점[16], 종합병원 간호사 90.42점[17], 가정전문 간호사 97.40점[18]에 비해 낮은 결과를 보였다. 그러나 Frommelt [35]의 도구개발 연구에서 임종간호태도의 평균이 2점 이상이면 긍정적이라고 해석한 점에 근거하여 본 연구대상자의 임종간호태도를 4점 만점에 계산하였을 때 평균 2.86점으로 긍정적으로 평가할 수 있다. 일반적 특성에 따른 임종간호태도는 총 임상경력이 3년 이상이고 최근 1년 이내 가족이나 지인의 죽음경험이 있고 임종관련 교육경험이 있는 경우, 임종간호태도 점수가 높았다. 이는 중환자실 간호사[16]와 종합병원 간호사[17]를 대상으로 임종간호태도를 조사한 연구에서 근무기간이 길고, 임종에 관련된 교육에 참여한 경험이 있거나 교육참여횟수가 많고, 근무기간 동안 죽음을 경험한 횟수가 많을수록 임종간호태도가 긍정적이었다는 결과와 유사한 결과이다. 가족이나 지인의 임종을 경험한 간호사가 긍정적인 임종간호태도를 보이는 것은 지인의 임종경험이 있는 호스피스 간호사가 긍정적인 임종간호태도를 나타낸 연구결과[37]와 유사한 맥락이다. 종합해보면, 임상경력이 길고 임종관련 교육을 받고, 주변 가족이나 지인의 죽음을 경험하면서 임종과정에 대한 지식과 이해도가 쌓이면서 간호사의 임종간호에 대한 태도가 좀 더 긍정적으로 성장할 수 있다. 따라서 임상경력과 교육경험, 다양한 임종경험을 바탕으로 간호사의 개별적 특성에 맞는 구체적인 임종관련 교육프로그램을 개발하고, 특히 임종간호태도가 부정적일 수 있는 취약한 간호사를 대상으로 프로그램을 적용할 필요가 있다. 또한, 임종간호 시뮬레이션 프로그램이 간호대학과 의과대학 학생의 임종간호태도에 긍정적인 영향을 미쳤다는 해외 선행연구[38]에 기초하여, 임종상황에 대한 경험이 부족하여 적극성이 부족하거나 부정적인 임종간호태도를 가질 수 있는 신규 의료진이 경력 초기부터 긍정적인 임종간호태도를 가질 수 있도록 적용할 수 있는 효과적이고 체계적인 시뮬레이션 프로그램의 개발이 필요하다.

본 연구에서 임종간호태도는 죽음인식, 삶의 만족도와 유의한 정적 상관관계가 있었다. 그러나 임종간호태도와 임종간호 스트레스는 유의한 상관성이 없는 것으로 나타났는데, 이는 중환자 간호사 대상[16] 연구결과와 유사한 맥락이다. 임종간호태도와 임종간호 스트레스 간의 상관성은 부적 상관관계[19]가 나타나기도 하고, 정적 상관관계[17]가 나타나기도 하여 개념 간의 상관성을 확정하기는 어렵다. 스트레스는 간호사의 소진경험을 촉진하고 탈진, 근무태만, 의욕상실[39] 등 부정적 태도와 연관될 수 있지만, 적정수준의 스트레스는 오히려 상황에 효과적으로 대응하도록 개인의 능력을 향상시키고 개인성장과 긍정적 태도함양을 강화할 수 있다. 그러므로 스트레스와 임종간호태도와의 관련성에 대해서는 좀 더 면밀하고 지속적인 반복연구가 필요한 것으로 생각된다.

본 연구에 참여한 간호사의 임종간호태도에 영향을 미치는 요인은 죽음인식, 3년 이상의 임상경력, 최근 1년 이내 가족이나 지인의 죽음경험, 삶의 만족도, 임종관련 교육경험 순으로 나타났으며, 이 다섯 가지 변인이 임종간호태도 변량을 34.0% 설명하는 것으로 나타났다. 죽음인식은 본 연구에 참여한 간호사의 임종간호태도에 가장 강력하게 영향을 미치는 요인이었다. 종합병원 간호사의 임종간호태도에 영향을 미치는 요인을 조사한 연구에서[17] 죽음인식 중 죽음의 긍정적인 의미와 생명존중의 의지가 높을수록 임종간호태도가 높았다. 가정전문간호사 대상 연구에서[18] 죽음인식 중에서도 특히 죽음에 대해 관심을 가지고 많이 생각하고 상상하는 죽음관여도가 높을수록 임종간호태도가 유의하게 높은 경향이 있었다. 간호사는 죽음을 반복적으로 겪고 임종상황에 노출이 많아지면서 죽음에 대한 수용도가 높아지고 죽음을 삶의 자연스러운 과정으로 받아들이게 된다[15]. 이는 간호사의 임종에 대한 전반적인 태도가 긍정적으로 발전하는 것을 의미하며[40], 선행연구와 본 연구의 결과에서 알 수 있듯이, 높은 죽음인식이 긍정적인 임종간호태도에 영향을 미친다. 그러므로 죽음에 대해 관심과 긍정적 인식을 높이고, 생명존중의지에 대한 이해도를 향상하는 교육프로그램은 긍정적인 임종간호태도를 촉진할 수 있을 것이다. 간호사들은 죽음에 대해 생각하고 이해하는 경험을 가질 수 있는 교육에 대한 요구도가 높았고[41], 이러한 요구도를 반영하는 감정돌봄 프로그램이나 임종체험 프로그램은 죽음에 대한 부정적 감정과 불안도를 감소시킬 수 있을 것으로 생각된다.

본 연구에서 삶의 만족도는 상급종합병원 간호사의 임종간호태도에 유의한 영향을 미치는 요인이었다. 삶의 만족도가 높은 사람들은 정서적으로 안정적이며, 스트레스 상황에 더 잘 대처하며[42], 긍정적이고 낙관적인 사고방식을 가지므로[43], 임종간호와 같은 감정적으로 힘든 상황에서도 긍정적인 태도를 유지하며, 임종간호상황에서 발생할 수 있는 다양한 어려움과 도전에 더 잘 대처하고, 환자와 가족에게 희망적이고 따뜻한 분위기를 조성할 수 있다. 선행연구에 따르면, 밀레니얼 간호사들은 간호근무환경이 만족스럽지 못하고, 간호직의 자율성이 낮을 때, 일과 삶의 균형이 만족스럽지 않다고 생각하며 높은 이직의도를 표현하였다[44]. 간호사의 이직의도는 간호 서비스의 질 저하로 이어질 수 있으며[44], 밀레니얼 세대와 Z세대 간호사가 일과 삶의 균형을 유지하고 삶의 만족도를 증진할 수 있도록, 자율성을 확립하고 업무특성을 개선하는 환경조성과 전략개발이 필요하다.

본 연구는 일개 상급종합병원을 대상으로 수행되었고, 보건의료인력실태조사[32]에 포함된 간호사의 특성과 일부 차이나는 특성분포를 나타내었음으로 연구결과를 일반화하기에는 한계가 있다. 이러한 제한점에도 불구하고 본 연구는 상급종합병원 간호사의 죽음인식과 삶의 만족도가 임종간호태도에 미치는 영향을 파악하였고, 임종간호 교육경험과 지인의 임종경험, 임상경력에 따른 임종간호태도의 차이를 확인함으로써 긍정적 임종환자 간호를 위한 시스템 개발의 기초자료를 제공하였다는 점에서 의의가 있다. 본 연구결과를 바탕으로 간호사의 개별적 특성에 맞는 임종관련 교육프로그램 개발이 필요하며, 긍정적인 죽음인식 함양과 부정적 죽음인식 개선을 위한 감정돌봄 프로그램과 임종체험 프로그램이 도움이 될 것으로 생각된다. 임종간호 스트레스와 임종간호태도 간 관계에 대한 지속적인 반복 연구가 필요하며, 간호사의 삶의 만족도를 높이기 위해 근무환경 개선과 자율성 확립을 통해 일과 삶의 균형을 보장하는 것이 필요하다. 연구결과의 일반화를 향상하기 위해 국내 다양한 지역의 병원에서 근무하는 간호사를 대상으로 후속 연구가 필요하다.

## 결론

본 연구에 포함된 간호사의 임종간호태도는 비교적 긍정적이고, 총 임상경력이 3년 이상이고, 최근 1년 이내 가족이나 지인의 죽음을 경험하였고, 임종관련 교육경험이 있는 경우, 임종간호태도가 더 긍정적이었다. 따라서 임상경력이 3년 미만이고, 임종관련 교육경험이 없는 간호사를 중심으로 개별적 특성에 맞는 구체적인 임종간호관련 훈련프로그램을 제공하는 것이 필요하고, 다양한 임종상황에 대한 시뮬레이션 교육프로그램은 임종간호태도 향상을 위해 고려해 볼 수 있을 것으로 생각된다. 높은 죽음인식과 삶의 만족도는 긍정적인 임종간호태도와 관련되며 죽음에 대해 관심을 가지고 긍정적으로 인식하고 성숙하게 반응할 수 있도록 돕는 감정돌봄 프로그램과 다양한 임종상황에 대응할 수 있는 임종체험 프로그램의 활용이 효과적일 수 있다. 특히 MZ세대 간호사의 삶의 만족도 향상을 위해, 일과 삶의 균형을 유지할 수 있도록 간호 근무환경을 개선하는 것이 필수적이다. 본 연구에서 임종간호 스트레스는 임종간호태도와 유의한 관련성이 없는 것으로 나타났으나 기존 연구에서의 관련성을 고려하여 추후 대규모 반복연구를 통해 개념 간 상관성을 확인해 볼 필요가 있다. 본 연구는 상급종합병원 간호사의 임종간호태도와 삶의 만족도와의 관련성을 확인하였고 임종간호 교육이 임종간호태도에 긍정적 영향을 미치며, 3년 미만의 간호사에게 임종간호태도 향상을 위한 적극적 개입이 필요함을 확인하였다는 측면에서 그 의의가 있다. 추후 부정적 임종간호태도에 특히 취약한 간호사 중심으로 긍정적 임종간호태도를 위한 개별화된 프로그램 개발과 효과검증을 제언한다.

## Figures and Tables

**Table 1.** Participants’ Characteristics and Attitudes toward End-of-life Care (N = 150)

Variables	Categories	n (%)	Attitudes toward end-of-life care	
			M ± SD	t or F (*p*)
				Post hoc^†^
Gender	Men	21 (14.0)	82.95 ± 8.87	-1.69 (.094)
	Women	129 (86.0)	86.26 ± 8.27	
Age (yr) (29.91 ± 3.71)	20∼<30	78 (52.0)	84.99 ± 8.49	1.48 (.231)
	30∼<40	69 (46.0)	86.43 ± 8.31	
	40∼<50	3 (2.0)	92.33 ± 5.51	
Marital status	Single	100 (66.7)	85.10 ± 7.72	-1.18 (.241)
	Married	47 (31.3)	87.02 ± 9.81	
Religion	No religion	108 (72.0)	85.52 ± 8.32	0.54 (.591)
	Religion	41 (28.0)	84.42 ± 7.34	
Education	College graduate	3 (2.0)	84.00 ± 4.58	1.35 (.262)
	Bachelor’s degree	122 (81.3)	85.34 ± 8.02	
	Master’s degree or higher	25 (16.7)	88.28 ± 10.22	
Total clinical career (yr)	1∼< 3^a^	23 (15.3)	79.04 ± 9.60	4.89 (.001)
	3∼< 5^b^	32 (21.3)	87.31 ± 6.57	a < b,c,d,e^†^
	5∼< 7^c^	34 (22.8)	87.26 ± 6.99	
	7∼< 10^d^	41 (27.3)	85.78 ± 7.32	
	≥ 10^e^	20 (13.3)	86.65 ± 10.65	
Current work unit	Ward	106 (70.7)	86.33 ± 7.65	1.08 (.285)
	Intensive care unit	44 (29.3)	84.52 ± 9.96	
Experience of an acquaintance’s death within 1 year	Yes	41 (27.3)	89.68 ± 8.38	3.61 (< .001)
	No	109 (72.7)	84.34 ± 7.97	
Experience of education about terminal care	Yes	53 (35.3)	89.19 ± 8.06	3.81 (< .001)
	No	97 (64.7)	83.95 ± 8.04	
Frequency of terminal care within 1 year	< 5	83 (55.3)	84.37 ± 8.80	2.76 (.067)
	5∼< 10	40 (26.7)	87.43 ± 8.28	
	≥ 10	27 (18.0)	87.78 ± 6.57	

M = Mean; SD = Standard deviation.

† Post hoc = Scheffé.

**Table 2.** The Levels of Death Perceptions, Terminal Care Stress, Life Satisfaction, and Attitudes toward End-of-life Care (N = 150)

Variables	Number of items	Scale range	Min	Max	Total scores	Average scores
					M ± SD	M ± SD
Death perceptions	36	1∼5	73	157	108.69 ± 13.14	3.02 ± 0.36
Terminal care stress	40	1∼5	40	200	153.14 ± 30.36	3.83 ± 0.76
Life satisfaction	1	1∼5	1	5	3.25 ± 0.72	3.25 ± 0.72
Attitudes toward end-of-life care	30	1∼4	65	109	85.80 ± 8.40	2.86 ± 0.28

Min = Minimum; Max = Maximum; M = Mean; SD = Standard deviation.

**Table 3.** Correlations between Death Perceptions, Terminal Care Stress, Life Satisfaction, and Attitudes toward End-of-life Care (N = 150)

Variables	1	2	3	4
	r (*p*)			
1. Attitudes toward end-of-life care	1			
2. Death perceptions	.41 (< .001)	1		
3. Terminal care stress	-.11 (.173)	-.05 (.523)	1	
4. Life satisfaction	.24 (.003)	.09 (.269)	.03 (.683)	1

**Table 4.** Factors Influencing Attitudes towards End-of-life Care (N = 150)

Variables	B	SE	β	t	*p*	95% CI		Tolerance	VIF
						lower	upper		
(Constant)	45.93	5.60		8.19	< .001	34.97	57.13		
Total clinical career (yr)									
1∼< 3 (ref.)									
≥ 3	5.76	1.59	.25	3.63	< .001	2.12	9.37	.96	1.04
Experience of an acquaintance’s death within 1 year (yes)	4.06	1.24	.22	3.17	.002	1.37	6.86	.96	1.04
Experience of education about terminal care (yes)	2.71	1.24	.16	2.19	.030	0.31	5.14	.89	1.12
Life satisfaction	2.28	0.79	.20	2.90	.004	0.82	3.89	.98	1.03
Death perceptions	6.55	1.63	.28	4.02	< .001	3.30	9.56	.90	1.12
R^2 ^= .36, Adjusted R^2 ^= .34, F = 15.95, *p < *.001, Durbin-Watson = 1.45									

SE = Standard error; CI = Confidence interval; VIF = Variance inflation factor; ref. = Reference.

## Data Availability

Please contact the corresponding author for data availability.
